# Identification of Major Risk Factors and Non-linear Effects to the Development of Left Ventricular Hypertrophy in Chronic Kidney Disease by Constructing and Validation of Nomograms

**DOI:** 10.3389/fmed.2022.914800

**Published:** 2022-07-13

**Authors:** Zhongcai Wu, Mengxia Shi, Le Wang, Ying Yao

**Affiliations:** ^1^Department of Nephrology, Tongji Hospital, Tongji Medical College, Huazhong University of Science and Technology, Wuhan, China; ^2^Department of Nutrition, Tongji Hospital, Tongji Medical College, Huazhong University of Science and Technology, Wuhan, China

**Keywords:** ventricular hypertrophy, chronic kidney disease, risk factor, nomogram, predictive model

## Abstract

**Background:**

Left ventricular hypertrophy (LVH) is a common cardiovascular complication among chronic kidney disease (CKD) patients. The present study aimed to identify major independent risk factors and determine their contribution and relationship to LVH development.

**Methods:**

Clinical and echocardiographic data of 2002 pre-dialytic CKD patients were retrospectively collected. Independent risk factors for LVH were identified using univariable and multivariable logistic regression. Nomograms together with restricted cubic splines method were employed to explore the effect size and possible non-linear relationship with regard to LVH. A simplified predictive model was constructed and its predictive ability was validated to demonstrate to which extent the identified risk factors accounted for LVH risk.

**Results:**

Multivariable logistic regression identified age, body mass index (BMI), systolic blood pressure (SBP), eGFR and hemoglobin as independent influencing factors for LVH. Nomogram revealed BMI, SBP and hemoglobin concentration as the most important risk factors. Impaired renal function only showed obvious risk for LVH when eGFR declined below 30 ml/min/1.73 m^2^. Significant threshold effects existed for blood pressure and obesity that the risks for LVH doubled when SBP exceeded 160 mmHg or BMI exceeded 30 kg/m^2^. The predictive model constructed performed well on both the training and validation cohort using calibration curve, ROC curve and AUC value, with AUC above 0.80 for both the training cohort and the validation cohort.

**Conclusions:**

With the help of nomogram model, we identified five independent factors that explain a large proportion of LVH risk in CKD patients. Among them, major contribution to LVH development was resulted from comorbidities and complications of CKD (hypertension, anemia, obesity) rather than eGFR reduction *per se*. Non-linear relationship and threshold relationship between eGFR, blood pressure, obesity and LVH risk were also identified.

## Introduction

Ventricular hypertrophy is the structural reconstruction of the heart that closely associate with cardiac function (1, 2). The Framingham cohort study has confirmed the role of ventricular hypertrophy as a harbinger for all-cause mortality and mortality from cardiovascular disease (3). Thus, ventricular hypertrophy is a good indicator reflecting the present cardiovascular risks.

In patients with chronic kidney disease (CKD), cardiovascular complications are major cause for morbidity and mortality, and the hazards for adverse cardiovascular events or cardiac mortality are greatly increased even in patients with mild renal function decline or proteinuria (4–6). Accordingly, the prevalence of ventricular hypertrophy is considerably elevated in patients with CKD (7). The development of ventricular hypertrophy is a gradual cumulative process that result from the persistent damaging of underlying risk factors (8). However, as CKD is a complex syndrome involving a variety of pathological statuses, i.e., low renal clearance, anemia, hypertension and disturbances of water and electrolyte balances, the relative contribution of each pathophysiological changes to the development of ventricular hypertrophy has not been comprehensively evaluated.

Nomograms are risk assessment tools that visually display the contribution of each risk factor (9). Together with restricted spline method to simulate non-linear relationships (10), the nomogram model could demonstrate detailed relation between risk factors and the outcome of interest. Furthermore, as a method for risk prediction, reliable methods for validating the performance of nomogram models exist that allow us to testify the correctness of our hypothesis. To our knowledge, nomograms predicting risks for ventricular hypertrophy in CKD patients have not been developed thus far. In this study, we retrospectively collected data from 2,002 CKD patients, and dividing 1,083 of them into the training cohort and 919 into the validation cohort. Independent risk factors for development of ventricular hypertrophy were assessed from the training cohort and corresponding nomogram was constructed for intuitive presentation of relative contribution and detailed relation, and the predictive accuracy of the nomogram was verified in both the training and the validation cohort.

## Materials and Methods

### Study Population

Eligible patients comprise adults who were diagnosed as CKD, not on renal replacement therapy (RRT) and had echocardiographic measurements from August, 2013 to June, 2022 in Tongji Hospital, Tongji Medical College of Huazhong University of Science and Technology (Wuhan, China). CKD diagnosis complied with the KDIGO 2012 CKD guideline (11), which stated persistent decreased eGFR <60 ml/min/1.73 m^2^, urine abnormalities or histological or imaging renal abnormalities for more than 3 months. Exclusion criteria included: (1) prior RRT treatments; (2) acute on chronic kidney disease; (3) active malignances; (4) congenital heart disease; (5) women who were in pregnancy or perinatal stage (<6 weeks postpartum). The study was approved by the medical ethical review board of Tongji Hospital, Tongji Medical College, Huazhong University of Science and Technology and was conducted according to the Declaration of Helsinki. Informed consent was waived due to its retrospective design and observational nature.

### Data Collection

We collected data from electronic medical record systems consisting of: (1) General characteristics and medical histories including sex, age, birthdate, main diagnoses; past histories of congenital heart diseases, diabetes mellitus and hypertension; blood pressure, body weight and height; (2) echocardiographic measurements including left ventricular internal diameter in diastole (LVIDD), the diastolic thickness of the interventricular septum (IVST) and left ventricular posterior wall thickness (PWT); (3) laboratory results including the blood routine test (white blood cell count and composition, red blood cell count, hemoglobin, platelet); alanine transferase (ALT), aspartate transferase (AST), blood albumin (Alb), creatinine (Cr), urea, uric acid (UA), bicarbonate, total cholesterol and triglyceride, blood glucose and Glycated hemoglobin (HbA1c); blood parathyroid hormone (PTH), 25-hydroxyvitamin D [25(OH)VitD], erythrocyte sedimentation rate (ESR); (4) urine measurements including 24 h urine volume and 24 h urine albumin excretion (24 hUalb). eGFR was calculated by the creatinine-based Chronic Kidney Disease Epidemiology Collaboration (CKD-EPI) equation as follows (12):

for females with creatinine ≤ 0.7 mg/dL,
  $$\begin{array}{l} eGFR=144\times {\left(creatinine÷0.7\right)}^{-0.329}\times 0.993^{Age} \end{array}$$
for females with creatinine > 0.7 mg/dL,
  $$\begin{array}{l} eGFR=144\times {\left(creatinine÷0.7\right)}^{-1.209}\times 0.993^{Age} \end{array}$$
for males with creatinine ≤ 0.9 mg/dL,
  $$\begin{array}{l} eGFR=141\times {\left(creatinine÷0.9\right)}^{-0.411}\times 0.993^{Age} \end{array}$$
for males with creatinine > 0.9 mg/dL,
  $$\begin{array}{l} eGFR=141\times {\left(creatinine÷0.9\right)}^{-1.209}\times 0.993^{Age} \end{array}$$

CKD staging was conducted according to the KDIGO 2012 guideline (11) that divided patients with confirmed CKD diagnosis into the following categories based on eGFR: stage 1 (eGFR ≥ 90.0 ml/min/1.73 m^2^); stage 2 (eGFR 60.0 ~ 89.9 ml/min/1.73 m^2^); stage 3 (eGFR 30.0 ~ 59.9 ml/min/1.73 m^2^); stage 4 (eGFR 15.0 ~ 29.9 ml/min/1.73 m^2^); stage 5 (eGFR <15.0 ml/min/1.73 m^2^).

### Definition of Left Ventricular Hypertrophy

Left ventricular mass (LVM) is calculated by the following formula based on echocardiographic parameters (13):


$$\begin{array}{l} LVM=0.81\times 1.04\times \left({\left(LVIDD+IVST+PWT\right)}^{3}-LVIDD^{3}\right)\\ \;+0.6 \end{array}$$

Left ventricular mass index (LVMI) reflects the extent of ventricular hypertrophy:


$$\begin{array}{l} LVMI=LVM÷height^{2.7} \end{array}$$

Left ventricular hypertrophy (LVH) was diagnosed in males by *LVMI*>49.2 *g*/*m*^2^, and in females by *LVMI*>46.7 *g*/*m*^2^.

### Determination of the Training and Validation Cohort

The study population was split into 2 parts mainly by the time of examination. All the patients of the training cohort underwent echocardiographic examination before 2019, while more than 90% of patients in the validation cohort underwent echocardiographic examination after 2019. The remaining less than 10% of patients in the validation cohort had clinical visit after 2019 and were thus included in this group.

### Statistical Analysis

Categorical variables were represented as number (percentage). Continuous variables were either represented as mean ± standard deviation when normally distributed or as median (25th ~ 75th interquartile value) otherwise. Independent *t* test or Mann-Whitney U test was used according to data distribution to compare continuous variables. Chi-square test was conducted to compare nominal variables. Ordinal variables were compared using Mann-Whitney U test.

Risk factors for LVH were identified by logistic regression analysis. Significant factors in univariable logistic analysis were then fitted into multivariable logistic analysis using stepwise forward likelihood ratio (LR) method to select independent risk factors. Odds ratio (OR) and 95% confidence interval (95% CI) were used to describe risk factors.

Independent risk factors in above analysis were used to construct the initial nomogram model wherein restricted cubic spline method was employed to identify possible non-linear relationships between risk factors and LVH probability. In developing the final predictive model, variables with highly non-linear relationship with regard to LVH risk was converted to categorical variables, and other variables were fitted with a linear relationship to simplify the model. Consistency between the predicted and actual risk was compared by drawing calibration curves. Except the apparent performance of the predictive model in the training cohort, internal and external validation were also performed by bootstrap method and by inspecting performance in the validation cohort, respectively. Receiver operator curves were constructed in both the training and validation cohort to assess discriminative ability of the predictive model to separate patients with and without LVH. The Youden's index was used to estimate the cut-off point for discrimination, and sensitivity and specificity were calculated. Overall performance was estimated by AUC (area under curve) value of the ROC curves, and DeLong non–parametric method was used to compare AUC values in the training and validation cohort.

Double-sided *P* < 0.05 was considered as statistically significant in all analyses. Descriptive analysis, intergroup comparison and logistic regression was performed using IBM SPSS 26.0.0.0 (Chicago, IL, United States, IBM SPSS Statistics, RRID: SCR_019096). Nomogram, calibration curves and ROC Plots were constructed by R version 4.1.1 (R Project for Statistical Computing, RRID: SCR_001905) using the *rms* and *pROC* package.

## Results

### Characteristics of the Study Population

The study population consisted of 2,002 patients including 1,083 patients in the training cohort and 919 patients in the validation cohort. The mean age was 49.2 years and 55.6% of the population were men. Median eGFR value was 34.6 ml/min/1.73 m^2^, and CKD stage 1 to 5 constituted 17.5, 14.6, 21.8, 16.2, and 29.9% of the study population, respectively. Median urine albumin excretion was 1.22 g/24 h. The training cohort and the validation cohort were mainly separated temporally (all the participants in the training cohort were recruited before 2019 while the majority of the validation cohort were recruited since 2019). Compared with the validation cohort, the training cohort were older and had worse renal function, but were comparable in terms of blood calcium, phosphorus and urine albumin excretion. LVH was presented in 38.3% of the training cohort and 32.8% of the validation cohort. Detailed information was listed in Table 1.

**Table 1:** Characteristics of the study population. BMI, body mass index; SBP, systolic blood pressure; DBP, diastolic blood pressure; UALB, urine albumin; CKD, chronic kidney disease; LVH, left ventricular hypertrophy.

**Variables**	**Total (*n* = 2,002)**	**Training cohort** **(*n* = 1,083)**	**Validation cohort** **(*n* = 919)**	***P***
Age (years)	49.2 ± 14.9	50.5 ± 14.5	47.7 ± 15.2	<0.001
Sex (Male)	1,113 (55.6%)	622 (57.4%)	491 (53.4%)	0.072
BMI (kg/m2)	24.0 ± 3.8	23.8 ± 3.7	24.2 ± 3.9	0.029
SBP (mmHg)	140.7 ± 24.6	143.3 ± 24.7	137.7 ± 24.1	<0.001
DBP (mmHg)	87.5 ± 15.0	86.6 ± 14.7	88.7 ± 15.3	0.001
eGFR (ml/min/1.73 m2)	34.6 (12.4–73.0)	27.2 (11.8–60.5)	45.9 (14.2–84.7)	<0.001
Uric acid (μmol/L)	403 (329 ~ 486)	417 (340 ~ 499)	391 (321 ~ 471)	<0.001
Albumin (g/L)	38.5 (32.5 ~ 42.3)	39.0 (33.9 ~ 42.3)	37.9 (30.4 ~ 42.2)	0.001
Hemoglobin (g/L)	112.2 ± 27.2	109.8 ± 26.5	115.0 ± 27.8	<0.001
Calcium (mmol/L)	2.19 (2.06 ~ 2.31)	2.20 (2.06 ~ 2.31)	2.18 (2.05 ~ 2.30)	0.139
Phosphorus (mmol/L)	1.20 (1.02–1.44)	1.19 (1.01–1.44)	1.20 (1.03–1.45)	0.143
24 h UALB (g/24 h)	1.22 (0.42 ~ 2.80)	1.28 (0.43 ~ 2.73)	1.17 (0.40 ~ 2.92)	0.714
CKD stage				<0.001
Stage 1	350 (17.5%)	143 (13.2%)	207 (22.5%)	
Stage 2	292 (14.6%)	132 (12.2%)	160 (17.4%)	
Stage 3	437 (21.8%)	234 (21.6%)	203 (22.1%)	
Stage 4	324 (16.2%)	212 (19.6%)	112 (12.2%)	
Stage 5	599 (29.9%)	362 (33.4%)	237 (25.8%)	
LVH	716 (35.8%)	415 (38.3%)	301 (32.8%)	0.010

*BMI, body mass index; SBP, systolic blood pressure; DBP, diastolic blood pressure; UALB, urine albumin; CKD, chronic kidney disease; LVH, left ventricular hypertrophy*.

### Independent Risk Factors for LVH

We assessed risk factors by applying logistic regression model in the training cohort as shown in Table 2. Age, BMI, blood pressure, eGFR, hemoglobin, uric acid, bicarbonate, serum electrolyte concentration and lipid profiles, urine albumin excretion, ALT and diabetes were associated with LVH in univariable analysis. After fitting significant factors into multivariable logistic regression with forward LR variable selection, higher age, BMI and blood pressure were identified as independent risk factors for LVH while higher hemoglobin and eGFR was protective for LVH.

**Table 2:** Logistic regression analyses for LVH in the training cohort.

**Variables**	**Univariable model**		**Multivariable model**	
	**OR (95% CI)**	***P***	**OR (95% CI)**	***P***
Age/(10 years)	1.35 (1.23–1.47)	<0.001	1.18 (1.05–1.32)	0.004
BMI/(1 kg/m^2^)	1.09 (1.06–1.13)	<0.001	1.14 (1.09–1.19)	<0.001
SBP/(10 mmHg)	1.43 (1.34–1.52)	<0.001	1.30 (1.21–1.39)	<0.001
Hemoglobin/(10 g/L)	0.75 (0.71–0.79)	<0.001	0.81 (0.74–0.88)	<0.001
eGFR/(10 ml/min/1.73 m^2^)	0.77 (0.73–0.81)	<0.001	0.91 (0.85–0.97)	0.005
Bicarbonate/(1 mmol/L)	0.90 (0.88–0.93)	<0.001		
Uric acid/(100 μmol/L)	1.12 (1.02–1.24)	0.023		
Potassium/(1 mmol/L)	1.60 (1.33–1.93)	<0.001		
Natrium/(1 mmol/L)	1.08 (1.03–1.12)	0.001		
Chloride/(1 mmol/L)	1.06 (1.03–1.09)	<0.001		
Calcium/(1 mmol/L)	0.12 (0.06–0.24)	<0.001		
Phosphorus/(1 mmol/L)	4.47 (3.07–6.50)	<0.001		
Magnesium/(1 mmol/L)	5.31 (1.90–14.85)	0.001		
ALT/(10 U/L)	0.31 (0.14–0.70)	0.005		
TC/(1 mmol/L)	0.86 (0.80–0.92)	<0.001		
ln (24 h UALB)/(mg/24 h)	1.18 (1.08–1.30)	<0.001		
Diabetes	2.13 (1.58–2.87)	<0.001		

*BMI, body mass index; SBP, systolic blood pressure; ALT, alanine transferase; TC, total cholesterol; UALB, urine albumin. Multivariable model was conducted by fitting significant factors in univariable model using stepwise forward likelihood ratio method. Only factors with statistical significance were displayed. P < 0.05 was considered statistically significant*.

### Relationship Between Risk Factors and LVH

**Figure 1:**
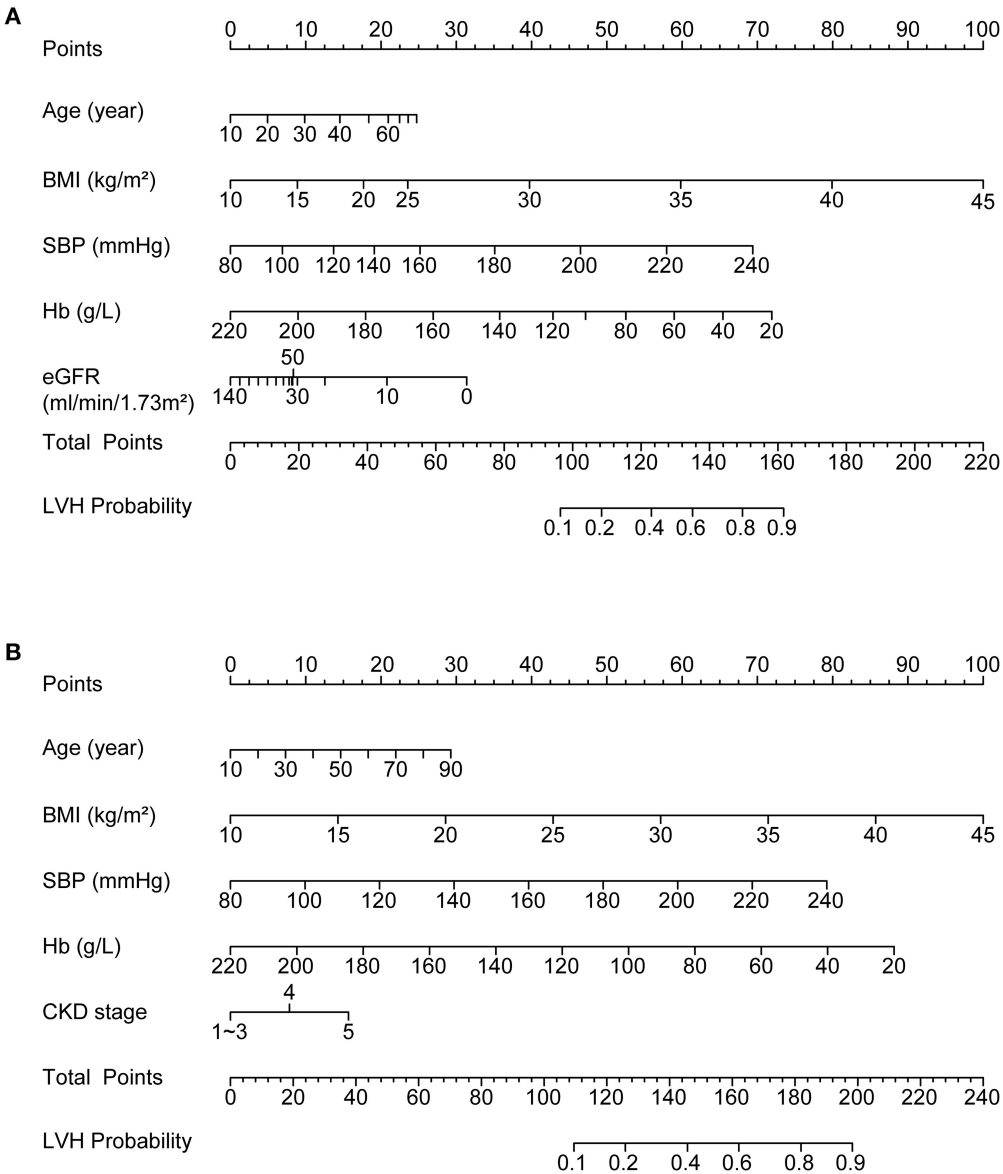
Nomograms depicting the relationships between independent risk factors and LVH probability **(A)** by incorporating restricted cubic spline method to investigate non-linear relationships; **(B)** with simplification as a final predictive model to improve ease of use. LVH, left ventricular hypertrophy.

To identify the relative contribution of risk factors to LVH as well as to assess possible non-linear relationship, we fitted the aforementioned independent risk factors into a nomogram model with the application of restricted cubic spline method to each factor. As shown in Figure 1A, the major contribution to LVH resulted from comorbidities or complications of CKD, i.e., hypertension, anemia and obesity, while reduced eGFR *per se* exerted a minor and obviously non-linear effect on LVH as risks greatly increased only when eGFR decreased below 30 ml/min/1.73 m^2^. Other factors displayed effects more closely resembling linear relationships, but significant threshold effect could be observed for SBP and BMI: as SBP exceeds 160 mmHg and BMI exceeds 30 kg/m^2^, the same degree of increasement thus far would result in doubled risk for LVH development compared to the range below the threshold value.

### Construction and Validation of the Predictive Nomogram Model

For simplicity as well as conformity with daily clinical practice, the non-linear relationship between eGFR and LVH were modeled by CKD stage and stage 1–3 were combined into a single category. All the other variables were approximated by linear relationship. The final nomogram predictive model was displayed in Figure 1B. Application of the nomogram tool simply requires finding the value of each variable on the corresponding axis, mapping the value to matched point on the top axis and then summing each point to get a total point. The risk of LVH could be determined by matching the total point to a probability on the bottom axis of the figure. Next, we assessed the performance of the predictive model by calibration curves and ROC curves in the training cohort as well as using a bootstrap method as internal validation and in the validation cohort as external validation.

### Measuring Accuracy of the Predictive Model by Calibration Curves

**Figure 2:**
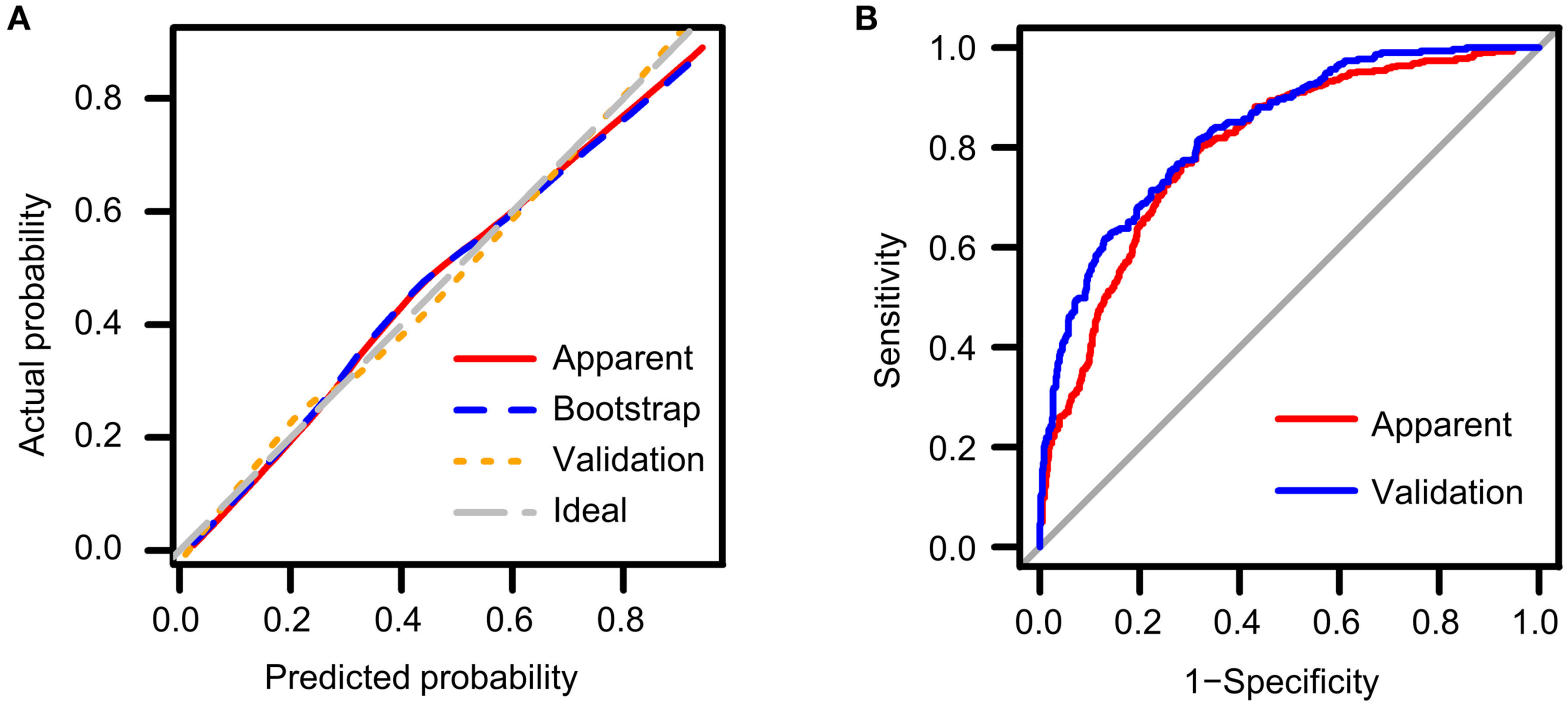
Validation of the predictive nomogram model **(A)** using calibration curves to reflect biases between ideal status and model performance; **(B)** using ROC curve to reflect discriminative ability of the model. Apparent: performance of the model in the original training cohort; Bootstrap: performance of the model in the bootstrap replaced population; Validation: performance of the model in the validation cohort.

Calibration curves measure how far the predictions of the model are from the actual observed outcomes. As shown in Figure 2A, the ideal model prediction was represented by the gray diagonal dashed line where predicted probability is completely matched to the actual situation. The distances between model prediction and this ideal line represents the accuracy of prediction. We assessed the model performance in the original training cohort (red solid line) as well as in the training cohort by bootstrap method (blue dashed line) and in the validation cohort (orange dotted line). In general, the three lines showed good accordance with respect to the ideal line, indicating good predictive ability especially for the validation cohort. However, in the original cohort we could observe a slight downward deviance in the high-risk range indicating overestimation and a slight upward deviance in the middle range indicating underestimation.

### Measuring Discriminative Ability by ROC Curves

ROC curves measure the ability of the predictive model to discriminate different outcomes, that is, the presence or absence of LVH in our study (Table 3). Best cut-off points for discrimination could be estimated using Youden's index where the sum of sensitivity and specificity maximize. General performance of the model was reflected as AUC value. We constructed ROC curves both for the training cohort (red line) and the validation cohort (blue line) as shown in Figure 2B. The AUC value was 0.801 (95% CI: 0.774 ~ 0.827) in the original cohort and 0.834 (95% CI: 0.808 ~ 0.861) in the validation cohort, and the two AUC values did not differ significantly, which confirmed that our model not only performed with reasonable accuracy but also had some generality. A probability of approximately 0.4 is suggested to be used to separate patients with and without LVH as reflected by the Youden's index, which resulted in a sensitivity of 0.766 and specificity of 0.717 in the training cohort, and a sensitivity of 0.817 and a specificity of 0.681 in the validation cohort.

**Table 3:** Summary of the performances of the predictive nomogram model.

	**Training cohort**	**Validation cohort**	***P***
Sensitivity	0.766	0.817	/
Specificity	0.717	0.681	/
Youden's index	0.483	0.498	/
AUC	0.801 (0.774–0.827)	0.834 (0.808–0.861)	0.078

*AUC, area under the curve*.

## Discussion

Ventricular hypertrophy is the morphological remodeling of the heart that resulted from long-term exposure to harmful cardiac damaging factors. Consequently, it could serve as a good indicator for patient prognosis, as this morphological change is closely related to adverse outcomes. Previous studies reported each 1 g/m^2.7^ increasement in LVMI would increase the risk for adverse cardiac events by 62%, while each 10% reduction of LVM by medical intervention could bring about 22% reduction in all-cause mortality risk and 28% reduction in risk of death due to cardiovascular events (14). Furthermore, ventricular hypertrophy is related to prolonged QT interval and increased risk of sudden death (15). Kidney disease has a close relationship with cardiac adverse events, as cardiovascular death is the leading cause of death in CKD patients with eGFR <60 ml/min/1.73 m^2^ and increases gradually as eGFR declines (16). Ventricular hypertrophy is a common morphological change of the heart caused by CKD which is classified as a typical feature of cardiorenal syndrome type IV (17). Thus, identifying main risk factors for LVH development in CKD patients and their relationship with LVH risk is paramount in preventing this complication and improving patient prognosis.

Former studies have reported elevated prevalence of LVH from the early stages of CKD (7, 15, 18). Mechanistically, LVH-inducing risk factors could be classified as three categories (19, 20): (a) pressure-related factors that mainly function by increasing the afterload of the heart, such as hypertension, arterial stiffness or vascular calcification; (b) volume-related factors that mainly function by increasing the preload of the heart, such as sodium and water retention, obesity, anemia and arteriovenous fistula; (c) load-independent factors that influences the metabolism, signal transduction or fibrosis of the heart, such as the activation of the renin-angiotensin-aldosterone system. As various complicated pathophysiological statuses coexist in CKD, the LVH-promoting effect is multifactorial in this special population. However, whether the elevated risk for LVH was mainly due to impaired eGFR *per se* or complications of CKD have not been determined.

In our study, we systemically screened for independent risk factors and fitted them into nomogram model in a multivariable way to allow for adjustment. Thus, we were able to find the contribution of each factor alone after removing major confounders. The nomograms intuitively reflect the contribution of each risk factor by drawing increasement of LVH risk directly caused by each unit increasement of the risk factor. By inspecting the resulting nomogram plot, we found reduced eGFR *per se* is not the major contributor to LVH development; instead, some commonly-seen comorbidities and complications of CKD, i.e., hypertension, anemia and obesity are major contributors to LVH. As impairment in renal function is hardly reversible in most CKD patients, this finding implies that major attention to prevent LVH should be diverted to ameliorating complications of CKD. This notion has been confirmed by studies reporting mitigation of LVH after dedicated control of hypertension and anemia (14).

Furthermore, incorporating the restricted cubic spline method in the nomogram model enables us to investigate non-linear relationships between risk factors and LVH. We found the effect of eGFR reduction is not evenly distributed among different stages of CKD, and only in patients with CKD stage 4 ~ 5 the effect for causing LVH by impaired eGFR became obvious. Thus, timely diagnosis and treatment of CKD to slow down the progression of eGFR decline might also be useful in preventing LVH development and reducing cardiovascular risks.

Other risk factors showed LVH-contributing effects more closely resembling linear relationships. However, for blood pressure and obesity, an apparent threshold effect existed that in SBP beyond 160 mmHg or BMI > 30 kg/m^2^, the contribution of each unit increasement would cause doubled effect compared to the situations below the threshold. In another words, lowering blood pressure or body weight control would result in more beneficial effects in highly hypertensive or obese patients. The existence of the threshold value justifies the concept of early intervention to meet the control target for blood pressure and obesity (21–23).

A simplified model was constructed with the aforementioned risk factors including age, SBP, BMI, CKD stage and hemoglobin. The performance of the predictive ability of the model to identify LVH in CKD patients were tested by both the calibration curve and the ROC curve. The predicted probability showed a reasonable conformity with the actual situation in the calibration curve and high AUC in the ROC plots, indicating that the above risk factors explained a large proportion of the underlying risks for LVH development in CKD patients and thus corroborating our identification of these factors as cardinal risk factors. Further, the performance was similar in the original training cohort, the replaced cohort using bootstrap method and the external validation cohort, suggests some degree of generality of the predictive model to estimate LVH risks in other populations. Youden's index suggested the cut-off point to discriminate between LVH and normal being set as 0.4 in our predictive model.

Our study has several limitations. First, we used LVH to estimate cardiovascular risks. However, as LVH is only an intermediate status of cardiac damage instead of hard outcomes of cardiovascular death or major adverse cardiovascular events, it could only serve as a partial substitute for estimating prognosis. Second, the cross-sectional design of the study limited its ability to make conclusions about causality, and the association we observed could only imply possible effectiveness in targeting these factors to prevent LVH development. Finally, our study recruited homogenous patients with the same ethnicity, which limited its extrapolation to other populations with different characteristics. Despite all these defects, our study also has some advantages: the multivariable adjustment allows us to observe the effects of each risk factor that are independent of major confounders; the employment of restricted cubic splines method enable us to explore non-linear relationship between risk factors and outcomes, and nomogram intuitively displayed the relative contribution of each factor that enabled us to assess the strengths of risk factors. Moreover, the verification of the performance of the predictive model reflects to which extent the above risk factors could account for the LVH risk, and the nomogram also gives us a handy tool to screen for patients with high cardiovascular risks.

In conclusion, we identified age, blood pressure, obesity, anemia and reduced eGFR as independent fisk factors for LVH development. We found that comorbidities of CKD but not impaired renal function *per se* was the major contributors to LVH; Instead, eGFR decline was only strongly correlated with LVH risk only when disease progressed to CKD stage 4 or beyond. Hypertension and obesity were highly risky for LVH development when SBP exceeds 160 mmHg and BMI exceeds 30 kg/m^2^. The construction of the nomogram offered a handy tool to predict patient LVH risk based on simple predictors.

## Data Availability Statement

The raw data supporting the conclusions of this article will be made available by the authors, without undue reservation.

## Ethics Statement

The studies involving human participants were reviewed and approved by the Medical Ethical Review Board of Tongji Hospital, Tongji Medical College, Huazhong University of Science and Technology. Written informed consent for participation was not required for this study in accordance with the national legislation and the institutional requirements.

## Author Contributions

ZW and YY designed the study. ZW and MS collected and organized data for analysis. ZW performed the statistical analysis and drafted the assay. LW checked the data and revised the draft. LW and YY supervised the study. All authors approved the final version of the manuscript.

## Funding

This work was financially supported by the National Natural Science Foundation of China (Grant No. 81974087 and 81873630).

## Conflict of Interest

The authors declare that the research was conducted in the absence of any commercial or financial relationships that could be construed as a potential conflict of interest.

## Publisher's Note

All claims expressed in this article are solely those of the authors and do not necessarily represent those of their affiliated organizations, or those of the publisher, the editors and the reviewers. Any product that may be evaluated in this article, or claim that may be made by its manufacturer, is not guaranteed or endorsed by the publisher.
